# Multi-Purpose Nanovoid Array Plasmonic Sensor Produced by Direct Laser Patterning

**DOI:** 10.3390/nano9101348

**Published:** 2019-09-20

**Authors:** Dmitrii V. Pavlov, Alexey Yu. Zhizhchenko, Mitsuhiro Honda, Masahito Yamanaka, Oleg B. Vitrik, Sergei A. Kulinich, Saulius Juodkazis, Sergey I. Kudryashov, Aleksandr A. Kuchmizhak

**Affiliations:** 1 Far Eastern Federal University, Vladivostok 690041, Russia; pavlov_dim@mail.ru (D.V.P.); g89leksig@mail.ru (A.Y.Z.); oleg_vitrik@mail.ru (O.B.V.); skulinich@tokai-u.jp (S.A.K.); 2 Institute of Automation and Control Processes, Far Eastern Branch, Russian Academy of Sciences, Vladivostok 690091, Russia; sikudr@sci.lebedev.ru; 3 Graduate School of Engineering, Nagoya Institute of Technology, Nagoya 466-8555, Japan; honda.mitsuhiro@nitech.ac.jp; 4 Graduate School of Engineering, Nagoya University, Nagoya 464-8603, Japan; yamanaka.masahito@h.mbox.nagoya-u.ac.jp; 5 Research Institute of Science and Technology, Tokai University, Hiratsuka, Kanagawa 259-1292, Japan; 6 Swinburne University of Technology, Hawthorn 3122 VIC, Australia; saulius.juodkazis@gmail.com; 7 Melbourne Centre for Nanofabrication, ANFF, Clayton 3168 VIC, Australia; 8 Lebedev Physical Institute, Russian Academy of Sciences, Moscow 119991, Russia; 9 National Research Nuclear University MEPhI, Moscow 115409, Russia

**Keywords:** direct femtosecond laser printing, nanovoid arrays, plasmonic sensors, refractive index and gas sensing

## Abstract

We demonstrate a multi-purpose plasmonic sensor based on a nanovoid array fabricated via inexpensive and highly-reproducible direct femtosecond laser patterning of thin glass-supported Au films. The proposed nanovoid array exhibits near-IR surface plasmon (SP) resonances, which can be excited under normal incidence and optimised for specific applications by tailoring the array periodicity, as well as the nanovoid geometric shape. The fabricated SP sensor offers competitive sensitivity of ≈ 1600 nm/RIU at a figure of merit of 12 in bulk refractive index tests, as well as allows for identification of gases and ultra-thin analyte layers, making the sensor particularly useful for common bioassay experiments. Moreover, isolated nanovoids support strong electromagnetic field enhancement at lattice SP resonance wavelength, allowing for label-free molecular identification via surface-enhanced vibration spectroscopy.

## 1. Introduction

Resonant oscillation of free electron plasma, surface plasmons (SPs), supported by either noble-metal nanostructures or metal-dielectric interfaces upon their excitation with visible or near-IR light is a key effect behind the realisation of state-of-the-art chemo- and bio-sensors. SPs give rise to highly-enhanced and localised electromagnetic (EM) fields, which strongly react on any perturbation of the surroundings [1], permitting detecting, for example, local changes of the refractive index (RI) caused by molecular binding, chemical reactions [2,3], and gases, as well as boosting the characteristic IR absorption and Raman scattering of the surrounding molecules [4,5]. These features made plasmonic-based label-free sensors extremely popular and valuable tools for medical diagnostics, environmental monitoring, food safety, and security [6].

Alongside with the significant progress in the field of SP sensors made in the past several decades, there is still a need for facile and inexpensive technologies allowing the fabrication of high-quality sensors having competitive characteristics and merging different modalities in a single sensor element [2,7,8]. Utilisation of well-developed top-down approaches as electron- or ion-beam milling allows the fabrication of plasmon-active nanostructures and related SP sensors at nanometre-scale precision. However, these approaches become extremely time- and money-consuming for the fabrication of mm-scale sensors. Noteworthy also is that single-use sensors are required for many applications, limiting the applicability of the mentioned methods for mass production. Various chemical synthesis methods are well adopted for mass production of only disordered plasmonic nanostructures of a certain size and shape [9,10]. Additional preliminary processing steps are required to arrange the synthesized nanostructures into the ordered arrays, which can support low-loss lattice-type SP resonances. Such geometrical resonances coming from periodical arrangement of coupled plasmonic nanoantennas can be characterized by a higher quality factor, which is indeed favourable for various sensing applications.

Alternatively, utilisation of direct pulsed laser processing of plasmon-active materials (e.g., noble metal films) can be considered as a promising route for high-performing flexible and inexpensive mass production of various plasmonic nanostructures and their arrangements [11,12,13]. However, the interaction of ultra-short femtosecond (fs) laser pulses with the metal films is typically associated with the ejection and redeposition of multiple ablative nanoparticles precluding clean and highly-reproducible printing of nanostructure arrays supporting spectrally-narrow SP resonances. The issue becomes even more challenging when ultra-fast direct laser printing is applied to fabricate nanostructure arrangements separated by micron-scale distances to bring the collective resonance to the near IR spectral range. The presence of transparency windows of aqueous solutions [14], as well as strong electromagnetic field localization supported by plasmonic nanostructures [15] make this spectral range extremely promising for multi-purpose SP sensor realization.

In this paper, we demonstrate a multi-purpose SP biosensing platform based on periodically-arranged nanovoids produced via inexpensive direct ablation-free femtosecond (fs) laser patterning of thin glass-supported Au films. Such nanovoid arrays support near-IR lattice-type SP resonances, which can be tailored to enable specific sensing modalities by adjusting both the nanovoid geometric shape and arrangement. The fabricated SP sensor offers competitive sensitivity of ≈1600 nm/RIU at a figure of merit of 12 in typical bulk refractive index test experiments, as well as allows identification of gases and ultra-thin superstrate layers, making the sensor particularly useful for common bioassay experiments. Moreover, the isolated nanovoids support strong electromagnetic field enhancement at lattice SP resonance wavelength, allowing label-free molecular identification via surface-enhanced vibration spectroscopy.

## 2. Results

**Figure 1:**
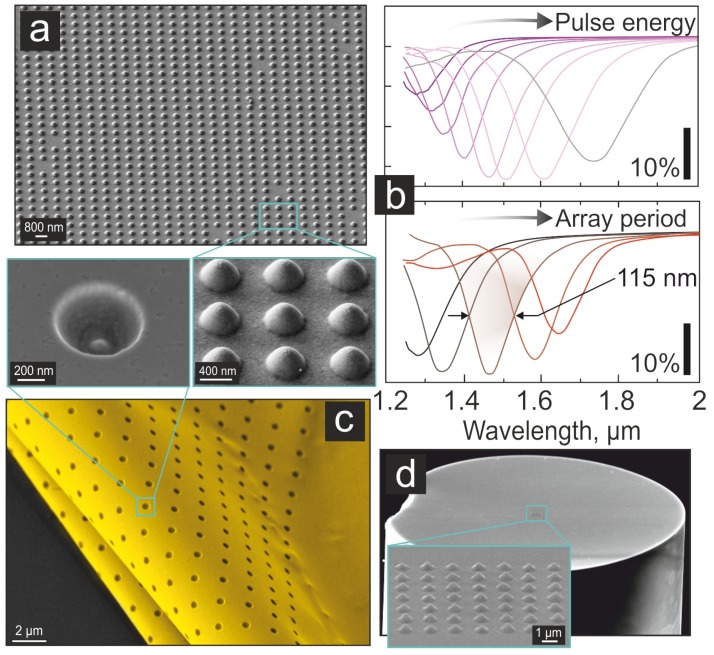
Nanovoid array SP sensor produced via direct ablation-free laser printing. (**a**) Side-view (view angle of $45^{\circ }$) SEM images of a nanovoid array printed at a 0.8-μm lattice periodicity (top); a close-up image of the isolated nanovoid backside and a close-up view of an array. (**b**) FTIR reflection spectra measured from the nanovoid arrays printed at a fixed periodicity of 1.2 μm and various pulse energies from 1–2.1 nJ (top); and at fixed pulse energy of 1.2 nJ and various periodicities from 0.8–1.2 μm (bottom). (**c**) SEM image (view angle of $78^{\circ }$) of the nanovoid array (the inset) produced at the end-face of a standard 125-μm diameter single-mode optical fibre. (**d**) SEM images showing the back-side of the Au film patterned by the nanovoid arrays.

SP sensors were fabricated via direct fs-laser ablation-free nanopatterning of the 50-nm thick Au film covering a silica glass substrate according to the procedure described elsewhere [16]. Briefly, the 200-fs second-harmonic (515 nm wavelength) laser pulses generated at a 200-kHz repetition rate by a regeneratively-amplified Yb: KGW laser system (Pharos, Light Conversion, Vilnius, Lithuania) were focused with a dry microscope lens (MY20X-804 Mitutoyo, Tokyo, Japan) having a numerical aperture of 0.42 to achieve a 1-μm-diameter Gaussian-shaped spot on the metal film surface. Within a certain range of applied energies, each laser pulse melted locally a small section of the irradiated Au film. The molten section of the film had weaker adhesion compared to the surrounding solid parts, resulting in its detachment from the supporting glass substrate via acoustic relaxation of thermal-generated stress and subsequent resolidification in the form of a parabolic nanovoid. A detailed description of the underlying physical process behind the formation of nanovoids can be found elsewhere [17,18]. The sample was translated by a three-axis nanopositioning system (Aerotech GmBH, Nurnberg, Germany) to fabricate rectangular nanovoid arrays by a single-shot single-structure approach (Figure 1a).

Ordered arrays of the laser-printed nanovoids demonstrated a pronounced dip in the near-IR reflection spectrum, further referred to as first-order lattice plasmonic resonance (FLPR), which can be explained in terms of the excitation and interference of SP waves [16,19]. In particular, periodically-arranged nanovoids with their smooth surface morphology provide efficient coupling of the normally incident IR radiation to the SP waves. Being simultaneously launched by these identical laser-printed nanostructures, SPs waves travel along the nanovoid curvy surface and further along the smooth Au film surface, interfering in between. In this respect, the spectral position of the FLPR resonance is defined by an “effective” lattice period, $p_{\mathrm{eff}}$, which depends on both the actual array periodicity *p*, as well as the geometric shape of the laser-printed nanostructures (nanovoids). The latter can be tailored by varying the incident pulse energy *E* (per single spot) [16]. These features provide remarkable flexibility for on-demand optimisation of the optical properties of the nanovoid arrays. In Figure 1b, two series of FTIR reflection spectra of the nanovoid arrays illustrate the tunability of the FLPR spectral position via tuning the geometry of nanovoids within the array printed at a fixed period p=1.1μm and variable pulse energy *E* (top panel, Figure 1b) and at a fixed pulse energy of E=1.3 nJ (nanovoid geometry) and array periodicity *p* ranging from 0.8–1.2 μm (bottom panel, Figure 1b).

In general, the described simple inexpensive and ablation-free fs-laser nanopatterning allows producing high-quality nanovoid arrays, which support geometry-dependent tunable near-IR SP lattice resonances with a Q-factor up to 13 (full width at half maximum of FLPR ≈ 115 nm; Figure 1b, bottom panel). Taking into account the high short-term pulse-to-pulse stability (≈0.05 %) of the laser system providing nearly perfect replication of the nanovoid shape, the resulting reproducibility of the FLPR resonance position was always within ±50 nm, as it was measured for multiple samples fabricated at fixed values of *E* and *p*.

Noteworthy, multi-beam interference lithography, as well as various DOE-mediated beam multiplexing techniques can be adopted to achieve extremely fast patterning of the Au films with the nanovoid structures at a printing rate up to 10${}^{6}$ elements per second [19,20,21,22,23,24]. Notably, the developed ablation-free fabrication protocol did not perturb the integrity of the Au film and its basic mechanical properties. The produced nanovoid array element was as robust as the initial unpatterned Au film on a substrate and was more durable than the lithographically-produced plasmonic nanoantenna array. Moreover, nanovoid arrays can be directly patterned on any smooth surface of a thin noble-metal film covering a heat-insulating substrate (e.g., glass or silicon). For example, such a structure can be produced within smooth microfluidic channels or even at the end-face of the optical fibre (Figure 1c). Moreover, using a standard procedure developed for 2D materials, the produced metal film patterned with the nanovoid arrays can be peeled off from the supporting glass (or silicon) substrate and transferred in the form of a membrane to another surface, including flexible polymers. Such a transfer does not cause any deformation of the nanovoids (Figure 1d), which gives extra versatility for the design and fabrication of functional sensing devices.

The spectral position of any plasmonic mode supported by a dielectric–metal interface is known to react on the RI of the dielectric media [1]. This feature makes the proposed laser-printed nanovoid arrays supporting the lattice-type FLPR with a rather high Q-factor in the near-IR spectral range potentially applicable for biosensing applications based on measurements of bulk and local variations of the RI [6]. To evaluate the performance of the produced sensors, we carried out three lines of experiments: (1) sensing bulk liquid filled above the arrays; (2) sensing thin films deposited on top of the arrays; and (3) measuring sensitivity to vapours and gases.

**Figure 2:**
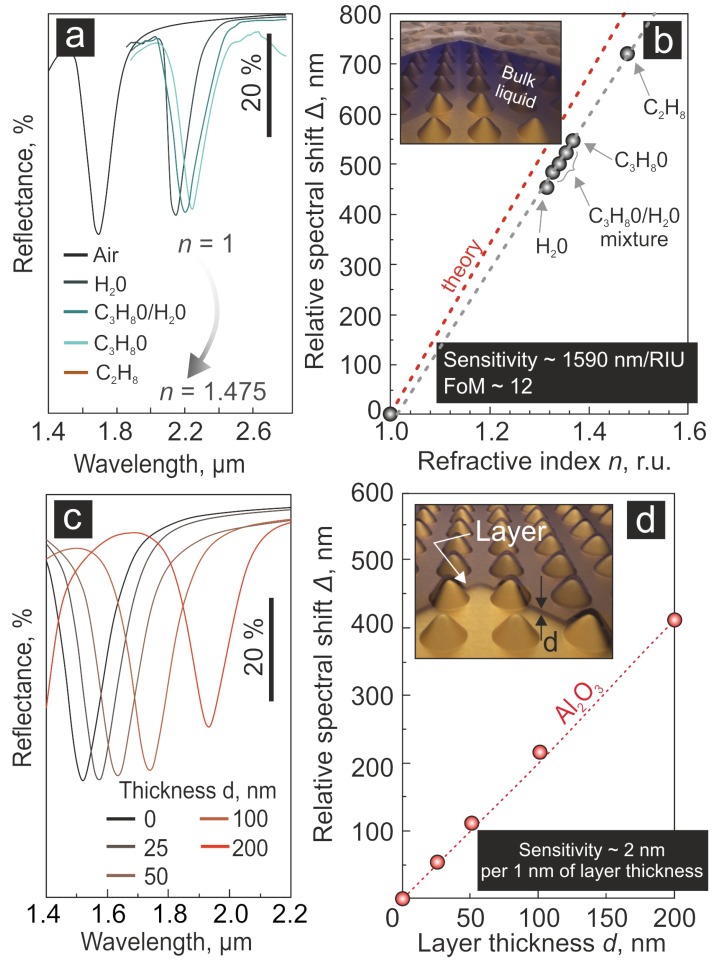
Performance of first-order lattice plasmonic resonance (FLPR) nanovoid array sensor for the detection of bulk and local refractive index changes. (**a**) FTIR reflection spectra showing the spectral position of the FLPR of the nanovoid array immersed in different liquids having refractive indices ranging from 1–1.475 (for toluene, the reflection spectrum is provided in Figure 3a). (**b**) Measured (markers) and calculated (red dashed line) relative spectral shift of the FLPR depending on the refractive index of the superstrate medium. (**c**) FTIR spectra of the nanovoid array covered by a thin alumina layer of variable thicknesses *d*. (**d**) Measured (markers) and calculated (dashed curve) relative spectral shift of the lattice resonance as a function of the thickness *d* of the alumina capping layer. The insets in Panels (**b**) and (**d**) illustrate the schematics of the experiments.

First, we assessed the sensitivity of the resonance of the nanovoid array to the variation of the bulk RI of the surrounding dielectric medium (superstrate). The experiments were performed by measuring the FTIR reflection from a fabricated nanovoid array with FLPR at around 1.7 μm, with various liquids filled in a home-built measuring cell having an IR-transparent quartz glass output window. We used water H${}_{2}$O, isopropanol C${}_{3}$H${}_{7}$OH, toluene C${}_{7}$H${}_{8}$, as well as water/isopropanol mixtures in various proportions, thereby achieving a set of RI in the range from 1 (no filling) to 1.475 (toluene) [14,25,26]. The corresponding FTIR reflection spectra (Figure 2a) demonstrated a clear redshift of the FLPR with an increase of the refractive index *n* of the superstrate medium. The dependence of the experimentally-measured relative spectral shift $\Delta =\lambda -\lambda_{0}$ of this lattice resonance on the superstrate bulk RI *n* shown in Figure 2b (markers) revealed its almost linear behaviour and demonstrated the relative sensitivity of ≈1600 nm/RIU with the figure-of-merit of about 12. Such competitive characteristics are good enough to detect the changes of the refractive index of the bulk dielectric superstrate as small as 10${}^{-5}$, taking into account the spectral resolution of the common FTIR spectrometers [27]. The trialled sensory elements withstood at least 50 measurement cycles in various liquids without changing the wavelength of the main lattice resonance in air, which confirmed the good mechanical stability and robustness of the isolated nanostructures within the array. The good mechanical stability of the nanovoids being combined with the excellent chemical stability of the Au material to various cleaning chemicals made the proposed sensor fully reusable.

In view of the nature of the FLPR at the patterned dielectric–metal interface, outlined above (see also [16,19]), the plasmon resonance was observed when the incident free-space wavelength $\lambda_{0}$ satisfied:

$$p_{\mathrm{eff}}=\lambda_{\mathrm{SP}}=\lambda_{0}\sqrt{\frac{1}{\varepsilon_{m}}+\frac{1}{\varepsilon_{s}}}$$

making this directly sensitive to the permittivity $\varepsilon_{s}$ of the environment. Given that the permittivity of gold $\varepsilon_{m}\gg \varepsilon_{s}$ in the IR range, the resulting dependence of the resonance on the refractive index $n_{s}$ was approximately linear,

$$\lambda_{0}\approx p_{\mathrm{eff}}n_{s}.$$

The observed linear slope of Δ(n) dependence (blue dotted line in Figure 2b) agreed well with the theoretically-predicted expectation (red dashed line in Figure 2b). A small deviation from the theory might be explained by an incomplete wetting of the metal surface with the superimposed liquids.

Second, we tested the performance of the nanovoid array with respect to a deposition of nm-thick layers, which was a rough imitation for chemical binding of analytes. Using the e-beam deposition, we imposed a series of alumina (Al${}_{2}$O${}_{3}$) layers ($n_{s}=1.742\pm 0.005$ at a 1.5–2-μm wavelength [28,29]) of a calibrated thickness *d* up to 200 nm above the nanovoid arrays. The resulting thickness of the deposited layers was verified using atomic-force microscopy, as well as, for thicker layers, zero-order optical transmission/reflection measurements. As expected for optically-thin superstrates, the recorded FLPR spectra demonstrated an approximately linear redshift Δ with an increase of the layer thickness d (Figure 2c,d). The presented data implied a sensitivity of an ≈2-nm spectral shift per 1-nm layer thickness, which would allow for the detection of sub-nm capping layers with conventional spectrometers (typically having a 1-nm spectral resolution).

Noteworthy, for layer thicknesses above 200 nm, the deviation between theoretical estimations and experimental results increased gradually (not shown here). This behaviour could be explained in terms of the complicated interaction of various waveguide and SPP modes supported by the air–dielectric–metal system having an optically-“thick” dielectric layer. The detailed analysis of the modes supported by such a system can be potentially performed using numerical methods. Nevertheless, the case of the thick capping layer was less relevant from the practical point of view, while such studies indeed fall outside the scope of this paper. We plan to analyse these features in a future study.

**Figure 3:**
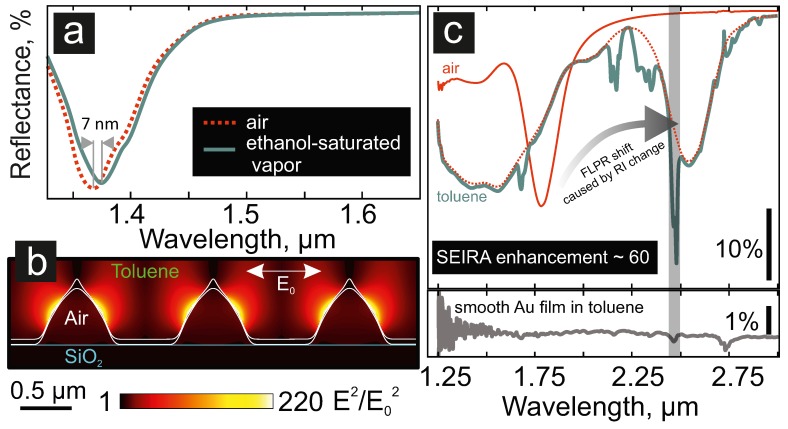
Various applications of the FLPR sensors. (**a**) Spectral response of the nanovoid array SP sensor caused by injection of saturated ethanol vapour. (**b**) Squared normalised EM-field amplitude E${}^{2}$/E${}_{0}^{2}$ calculated near the surface of the nanovoid array immersed in toluene, upon its excitation from the top by a linearly-polarised source at a 2.5-μm wavelength. (**c**) FTIR reflection spectra of the nanovoid array in air and under the toluene liquid layer. The dashed curve provides the contribution of the nanovoid array to the reflection spectrum, if taken without the absorption of toluene. The bottom panel shows the FTIR reflection from the smooth Au film surface covered by toluene obtained under the same conditions. SEIRA, surface-enhanced IR absorption

Third, we evaluated the performance of the proposed nanovoid array SP sensor towards the detection of gaseous environments. To this end, we exposed the sample to ethanol-saturated air in a home-made gas chamber setup previously described elsewhere [30,31]. The gas concentration in the chamber was calculated to be 7.8 vol.% (or ≈160 mg/L) according to the saturated vapour pressure, which was equal to 59.0 mmHg at 25 ${}^{\circ }$C. As shown in Figure 3a, such an exposure led to a detectable spectral shift of the FLPR by ≈7 nm via the corresponding change of the local RI of the surroundings. The FLPR spectral shift appeared to be caused by a two-fold effect, with a nanometre-thick ethanol layer absorbed onto the sensor surface, as well as an increase of the bulk refractive index caused by the gas presence. Importantly, the spectral shift was reversible when the chamber was refilled with air. Taking into account the spectral resolution of the FTIR system used (≈1 nm), as well as the observed spectral response of the nanovoid array to ethanol at its different concentrations, the detection limit achieved in this work was around 20 mg/L (4.3 × 10${}^{-4}$ mol/L). Though lower concentrations could not be detected with the proposed nanostructures, the results demonstrated here showed promise, while the detection limit of the sensing platforms can be improved via further optimization. This can be achieved, e.g., via fabrication of nanovoid arrays with their FLPR position <1 μm, where spectrometers with Si-based detectors (and a spectral resolution higher by an order of magnitude) can be applied. At the same time, optimization of the nanovoid geometry and chemical composition is also expected to push the sensitivity of the proposed structures towards lower gas detection limits, which is already a subject of our forthcoming studies.

Finally, we note that the nanovoids can produce strongly-enhanced and localised electromagnetic (EM) near-fields, which penetrated into the superstrate. We assessed this feature with the help of 3D FDTDnumerical simulations performed using a commercial software package (Lumerical Solutions). SEM images of the focused ion beam cuts previously reported in [17] were utilised to model the 3D shape of nanovoids. The nanovoid array placed below the semi-infinite layer of toluene was illuminated from the top with a linearly-polarised plane-wave source at the wavelength corresponding to the FLPR resonance in this environment. 3D simulations were performed at an ultra-fine 1-nm${}^{3}$ mesh considering periodic boundary conditions in both horizontal directions and perfectly matched layers as the boundary conditions limiting the vertical directions of the computation volume. Figure 3b shows the normalised squared EM-field amplitude E${}^{2}$/E${}_{0}^{2}$ calculated for the nanovoid array in toluene at a 2.5-μm incident wavelength, demonstrating up to a 220-fold enhancement. This feature makes the nanovoid arrays with tailored plasmonic response potentially applicable for label-free identification of molecular species based on surface-enhanced IR absorption (SEIRA). The FTIR reflection spectrum of the nanovoid array covered with an ≈10-μm-thick layer of toluene (Figure 3c, top panel) demonstrated an enhancement of several IR overtones of toluene, spectrally matching the FLPR of the array. In comparison to the same spectrum obtained from a smooth Au film (Figure 3c, bottom panel), this makes for an SEIRA enhancement factor of ≈60 for the certain IR band overtone of toluene.

## 3. Conclusions

In summary, in this paper, we demonstrated that laser-printed periodically-arranged nanovoid arrays offer a novel flexible multi-purpose sensing platform, potentially useful for bioassay studies and various chemo- and bio-sensing applications. Specifically, the FLPR of the nanovoid array was highly sensitive to both local and bulk RI changes of the superstrate. In addition, we also indicated the applicability of such arrays for gas and SEIRA-based molecular sensing.

Remarkably, even at moderate focusing conditions (NA = 0.5), fs-laser printing was capable of producing nanovoid arrays at sub-μm periodicity [32,33]. This will bring the FLPR spectral position to below 1 μm, where inexpensive and high-resolution spectrometers with Si-based detectors can be implemented. Moreover, as shown in previous studies, isolated nanovoids support tunable geometry-dependent localised plasmon resonances (LPRs) in the visible spectral range [32]. This makes it possible to utilise isolated nanovoids for molecule identification based on surface-enhanced Raman scattering, as well as LPR-based sensing, which is known to provide a better spatial resolution and better sensitivity to local RI variations, within the only sensor element. Noteworthy also was that the resonant nanovoid arrays can be produced on the surface of other noble metal films (as Ag, Cu, Pd, and Pt, [16]), as well as their alloys, providing flexibility in terms of tailoring their physical and chemical properties. This feature is expected to further extend the application range of the proposed SP sensors, for example to realize hydrogen gas sensors [34,35], etc.

Overall, a wide flexibility of the fabrication process with respect to nanovoid geometrical shape and array periods made it possible to adjust the spectral range of the proposed sensing platform suitably. Given the high speed and cost efficiency of the underlying liquid-free, chemical-free process, our design provides an important technological platform for realistic applications and devices for medical diagnostics, environmental monitoring, food safety, and security.
